# Visualizing Temperature Mediated Activation of Gelsolin and Its Deactivation By Pip_2_: A Saxs Based Study

**DOI:** 10.1038/s41598-017-04975-0

**Published:** 2017-07-05

**Authors:** Maulik D. Badmalia, Shikha Singh, Renu Garg, Ashish

**Affiliations:** CSIR-Institute Of Microbial Technology, Chandigarh, India

## Abstract

This is the first report describing temperature based initiation of gelsolin’s F-actin depolymerization activity, even in absence of free Ca^2+^ or low pH. Small angle X-ray scattering (SAXS) and circular dichroism (CD) studies revealed that temperature in the range of 30–40 °C is capable of opening the G1 domain alone, as remaining domains are held together by the Ca^2+^-sensitive C-tail latch without any loss in the secondary structural content. Full opening of all domains of tail-less gelsolin, and retention of closed shape for G2–G6 gelsolin merely by heating, further substantiated our findings. The Ca^2+^/pH independent activity of gelsolin near physiological temperature brought out a query: whether gelsolin is always active, and if not, what might deactivate it? Earlier, PIP_2_ has been reported to render gelsolin inactive with no structural insight. Reduction in shape parameters and modeling revealed that PIP_2_ reverses the temperature induced extension of g1-g2 linker leading to a compact shape seen for Ca^2+^-free gelsolin. Similar results for partially activated gelsolin (by low pH or Ca^2+^ ions below 0.1 μM) imply that inside cells, depolymerization, capping, and nucleation of F-actin by gelsolin is regulated by the culmination of local Ca^2+^ ion concentration, pH, temperature and PIP_2_ levels.

## Introduction

Shape-function studies on the six domain actin-assembly regulating protein, gelsolin has brought forth interesting findings, particularly how the compactly packed domains open up completely upon Ca^2+^ ions binding or partially by sensing low pH^1–4^. Gelsolin is one of the main members of the gelsolin family of proteins capable of depolymerizing F-actin by achieving active dissociation of actin units in assembly and keeping the dissociated actin units capped. Additionally, gelsolin can retard growth rate of F-actin filaments by binding to the growing end. In a seemingly contrasting manner, gelsolin can bind two monomeric or G-actin molecules, and can induce polymerization or nucleation of F-actin. This makes gelsolin as an important regulator of actin assembly inside cells. The plasma form of gelsolin is the fourth most abundant protein in plasma where in presence of 1 mM free Ca^2+^, plasma gelsolin performs the function of rapidly depolymerizing F-actin released upon cell death or injury, and keeps it from re-polymerizing. Latter ability has been attributed to potential role of gelsolin in different biomedical problems^5^. Thus, decoding the shape-function relationship of this varied function multi-domain protein has remained exciting to structure biologists. Crystallographic studies, radiolytic foot-printing and SAXS data analysis based models support that in absence of Ca^2+^ ions, the six homologous domains of this protein are packed tightly in a compact shape, and availability of Ca^2+^ ions induce systematic opening of the domains away from each other to expose actin binding sites^1, 2, 4^. Taking a cue from an earlier work which concluded that low pH can override the need for Ca^2+^ ions, our SAXS experiments revealed that buffer pH~5 can induce the first *i*.*e*. G1 domain to open up from other five domains, just enough to allow the actin binding site between the G1 and G2 domain to be available^3^. Additionally, scattering shape reconstruction of the full length gelsolin and its C-tail truncated version in low pH and/or in presence of Ca^2+^ ions clearly established that the C-tail latch which held together the G2 and G6 domains was sensitive only to Ca^2+^ ions and low pH could not open or affect the latch. In the same study, it was shown that addition of substantially less amount of Ca^2+^ ions to pH 5 activated gelsolin could induce complete opening of this six-domain protein analogous to that seen by 1 mM free Ca^2+^ ions at pH 8, representing plasma conditions which re-confirmed the Ca^2+^-specificity of the C-tail latch of gelsolin^2, 3^. The available biochemical and structural information summarizes that the compactly packed structure/shape represents inactive gelsolin, low pH can open G1 domain away from other domains which makes the protein partially active, and free Ca^2+^ ions can lead to a completely open structure/shape which can perform all stages of actin assembly regulation.

Interestingly, almost all the biophysical studies leading to above conclusions were done at temperatures below 37 **°**C, safely presuming that primary shape-function properties of gelsolin are regulated by only Ca^2+^ ions or low pH. Additionally, most of the crystal structures of gelsolin and its truncated form(s) +/− actin have been grown either at low pH or in temperature 4–24 **°**C (PDB ID/Temperature (°C): 1D0N/18, 1P8X/4, 1NPH/20, 1P8Z/20, 1RGI/4, 2FF3/20, 2FH1/4, 3FFK/24, 3FFN/24). Even our SAXS data collection of gelsolin −/+ Ca^2+^ and/or low pH were done at 10 °C^2, 3, 5^. In literature, we found only one study which experimentally compared the effect of temperature on functionality of gelsolin, *i*.*e*. how much Ca^2+^ ions are required by gelsolin to exhibit F-actin depolymerization activity^6^. These authors reported that at 37 °C, Ca^2+^-gelsolin depolymerization of pyrene labelled F-actin was detectable at 0.5 µM Ca^2+^ which reached half maximal at 2.2 µM Ca^2+^ and kept increasing till 0.1 mM Ca^2+^. In comparison, authors reported that at 24 °C depolymerization activity of gelsolin could be observed only at 10 µM Ca^2+^ which reached half-maximal 19 µM Ca^2+^ suggesting need for higher Ca^2+^ levels at lower temperature. Importantly, their results observed at 24 **°**C correlated well with previous reports done under similar conditions. This ten-fold difference in Ca^2+^ requirement at 24 *vs*. 37 **°**C clearly indicated that there is a role of increase in temperature in stabilizing the F-actin binding and depolymerizing competent conformations of gelsolin which overrides/complements the Ca^2+^-induced effects, though the authors mainly connected their findings to the role of Ca^2+^-sensitive C-tail region of gelsolin in enabling functional shape of full-length gelsolin and its variants.

Interestingly, a comparative study done for gelsolin and adseverin reported that with an increase of mere 10 °C in temperature the actin depolymerisation activity of gelsolin increased by 8 folds^7^. However, this increase in activity was again attributed to Ca^2+^ ion sensitive C-tail latch, as the dynamics of tail-less gelsolin matched with adseverin (the natural tail-less variant from gelsolin superfamily). In current work, while experimentally evaluating influence of temperature on F-actin depolymerization of gelsolin, we found that this protein can depolymerize F-actin when heated to temperatures close to 35 **°**C even in absence of Ca^2+^ ions or low pH *albeit* lesser than Ca^2+^-induced effects but comparable to influence of low pH. Experiments were repeated to confirm the observations followed by shape analysis using SAXS experiments which brought forward that the essential and minimal step to be depolymerization competent *i*.*e*. opening of G1 domain of gelsolin can be achieved by increase in temperature.

## Results

### Effect of Ca^2+^ ions/low pH and Temperature on depolymerization activity of gelsolin

**Figure 1:**
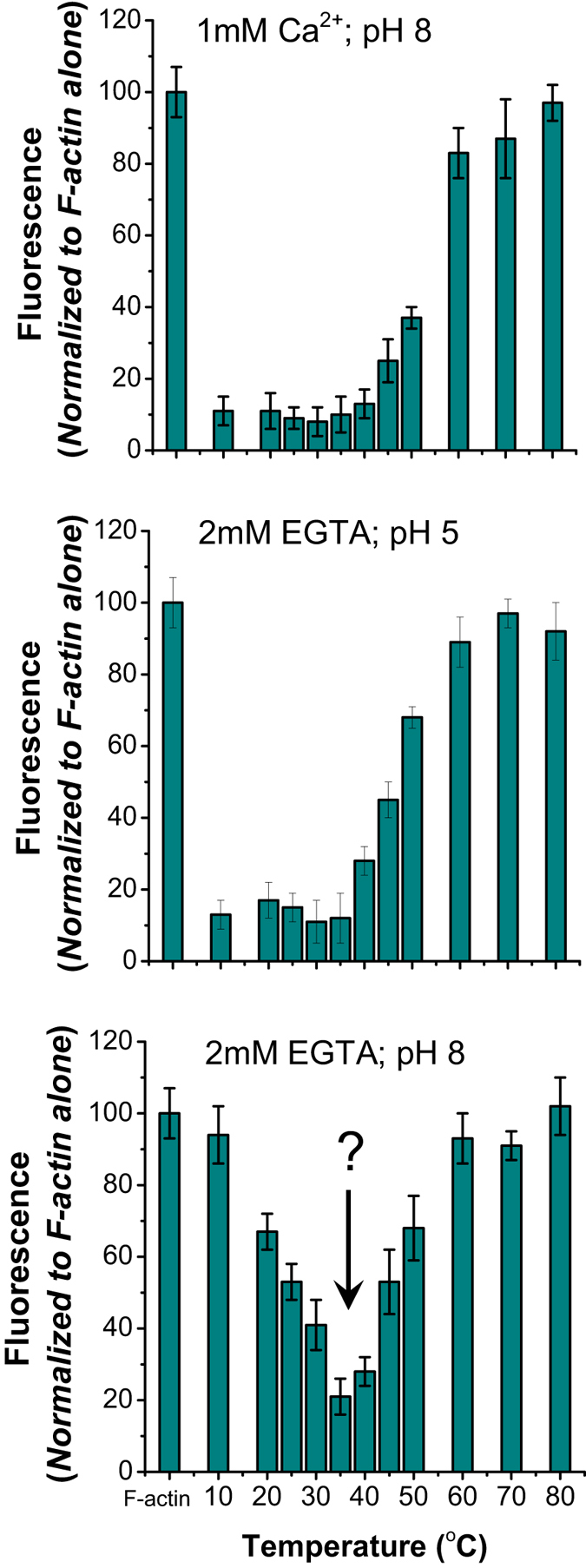
F-actin depolymerizing activities of gelsolin (at 0.83 μg/ml) are shown here by measuring relative decrement of pyrene fluorescence values as a function of temperature at which gelsolin in different buffers (mentioned in each panel) were heated to before performing the assays.

Earlier, using fluorescence based assays, it has been established that availability of Ca^2+^ ions or low pH can enable gelsolin to depolymerize labelled F-actin which can be recorded as a decrement in pyrene fluorescence^8^. Yet most of the previous experiments, in presence or absence of Ca^2+^ ions and pH close to 7.5 or at low pH in absence of Ca^2+^ ions were done at temperatures close to or below 25 **°**C, except one study where the depolymerizing activity of Ca^2+^-gelsolin was also compared at 37 **°**C^6^. To test dependence of F-actin depolymerization activity of gelsolin on temperature, heated samples of gelsolin, in buffers having 1 mM free Ca^2+^ (pH 8) (Fig. 1 top) and lacking any free Ca^2+^ ions (being chelated by excess EGTA) and pH 5 (middle) and pH 8 (bottom), were added to pyrene-labelled F-actin. In the control assays, only equivalent amounts of buffer were added, and all datasets were normalized to these read-outs from F-actin alone. Results showed that Ca^2+^-gelsolin heated up to 40 °C retained F-actin depolymerizing activity, but exposure to higher temperatures gradually reduced efficacy of Ca^2+^-gelsolin with almost complete loss by 60 °C (Fig. 1 top). Similarly, gelsolin at pH 5 displayed F-actin depolymerizing ability when heated till 35–40 **°**C, and then slowly efficacy of pH5-gelsolin decreased rapidly by 60 **°**C (Fig. 1 middle). Below, we have described the variable temperature SAXS and CD experiments which brought forth that gelsolin molecules start aggregating beyond 60 °C with clear observation of “whitish precipitation”. Interestingly, similar observations were reported in earlier studies^9, 10^. Thus, we considered that the loss of activity observed in the heated samples beyond 40 **°**C arise due to increasing fraction of activated gelsolin getting “consumed” in formation of heat induced non-functional aggregates.

To our sheer surprise, increasing the temperature of gelsolin in absence of Ca^2+^ ions and at pH 8, conditions known to keep gelsolin in inactive or non-depolymerizing form, led to decrement in fluorescence of pyrene-labelled F-actin. Repeated experiments provided similar results that as the temperature approached 35 **°**C, observation of decrement in fluorescence supported that somehow the increase in temperature from 10–35 **°**C induces a shape in gelsolin molecules which can depolymerize F-actin (Fig. 1 bottom). (*All experimental data shown in* Fig. 1 *are average of three independent experiments*). To the best of our knowledge, these experiments provided first direct indications that temperature alone can somehow allow gelsolin to achieve a shape which can depolymerize F-actin.

### Variation in SAXS data profile of gelsolin as a function of temperature

**Figure 2:**
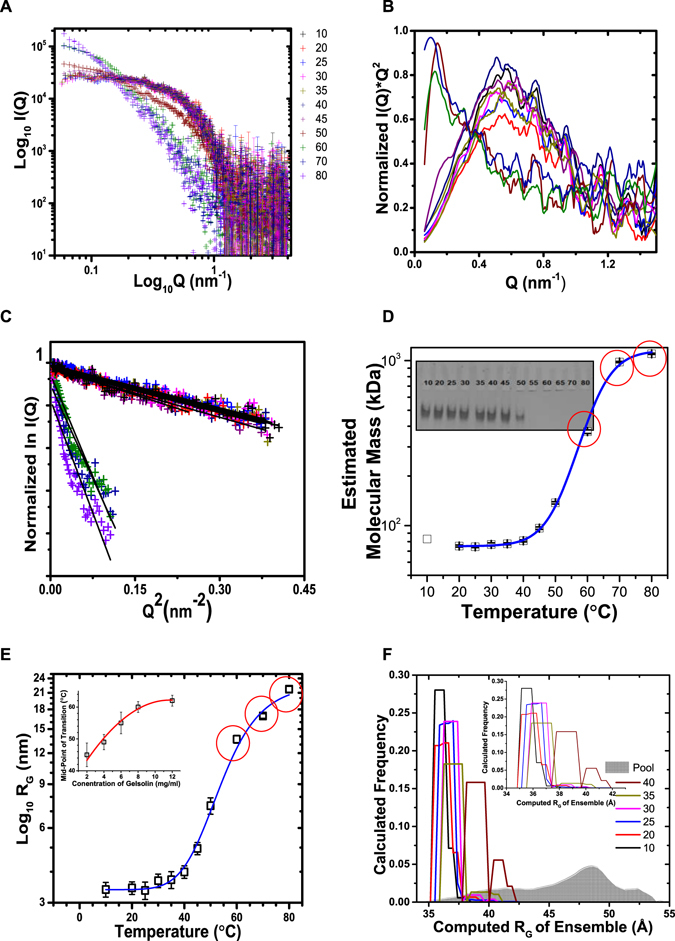
(**A**) The SAXS I(Q) profiles of gelsolin (8 mg/ml) at different temperatures in buffer lacking any free Ca^2+^ and pH 8 are plotted here. (**B**) This panel shows the Kratky plots of the SAXS datasets shown in panel (A). The smoothened versions were generated by averaging adjacent 5 data points). (**C**) The Guinier analysis of all the datasets presuming globular scattering profiles are shown here. (**D**) Variation in the molecular mass of the gelsolin molecules estimated using I_0_ values from Guinier analysis as a function of temperature are plotted here. The blue line represents the Sigmoidal fit to the data presuming two-state transition. Inset shows the image of the native PAGE of the heated samples. Red circles indicate samples where slight aggregation was visible and thus indicate uncertainty in estimation. (**E**) Variation in the R_G_ values as a function of temperature for gelsolin solution is plotted here (black squares). The blue line fitted through symbols shows the Sigmoidal fit to the observed trend in the increase of R_G_ values. The point of half-change for R_G_ values in samples varying in gelsolin concentration are shown in inset. (**F**) Variation in the calculated R_G_ values of the different conformations present in the ensemble as a function of temperature of experiment are presented against the pool of random conformations considered.

Figure 2A shows the SAXS I(Q) profiles acquired from a sample of full-length gelsolins as a function of temperature. In the double log plot, it was clear that initially the scattering particles or the protein adopted monodisperse scattering profile, which with increase in temperature increased in dimensions as evident from the elevated intensities at higher temperature. Peak-like profiles of the Kratky analyses of the SAXS datasets confirmed that gelsolin molecules in solution remain globular till 50 **°**C before associating into higher order aggregates as seen in the SAXS profiles collected at 60, 70 and 80 **°**C (Fig. 2B). The normalized Guinier approximation of the datasets also showed that there was slight increase in the average size of the predominant scattering species till 45–50 **°**C, followed by sudden increase in the average size of the scattering particles at 60–80 **°**C (Fig. 2C). To probe whether heating was inducing any soluble aggregates, samples of gelsolin treated to different temperatures were analyzed by native PAGE (Fig. 2D inset). The experiment carried out under non-denaturing condition indicated that gelsolin molecules primarily remained monomeric till 45 °C, before associating into larger entities which did not enter the native gel. *These observations correlated with the temperature variable SAXS and CD experiments described below*. Since, value of intensity at zero angle of scattering (I_0_) is directly proportional to square of the mass of the scattering species, and knowing that protein concentration remained comparable (*at least before onset of higher order aggregation*), we used I_0_ values estimated from Guinier approximations to estimate mass of the predominant scattering species in solution at different temperatures (Fig. 2D). The I_0_ values and the estimated molecular mass values along with their error are tabulated in Table 1. Guinier analysis profile indicated possible aggregation in the samples at and above 60 °C. As mentioned before and later, gelsolin samples at 60, 70 and 80 °C showed whitishness which brought in uncertainty in the estimation of molecular masses at these temperatures (*indicated as red circles in* Fig. 2D). Any case, we can conclude that the molecular mass estimation supported that the gelsolin molecules remained close to 75–80 kDa or monomer till 40 °C and there were some low order association in sample at 45 °C which could not be identified by native PAGE.

**Table 1:** Estimated molecular mass values of the scattering species in solution of gelsolin at different temperatures are tabulated below.

Temperature (°C)	Estimated I_0_ value (au)	Error in I_0_	Calculated Mass of scattering Species (kDa)	Equivalent Error in estimation (kDa)	Comments on sample
10	28559	552	(83)	—	
20	25890	362	75.2	1.1	Monomer
25	25602	259	74.4	0.8	Monomer
30	26444	579	76.9	1.7	Monomer
35	26686	387	77.6	1.1	Monomer
40	27847	385	80.9	1.1	Monomer
45	33202	561	96.5	1.6	Association
50	47278	785	137.4	2.3	Association
60	127729	3156	371.2	9.2	Aggregation
70	337280	2870	980.2	8.3	Aggregation
80	378500	3121	1100.0	9.1	Aggregation

The I_0_ values have been estimated from Guinier analysis of the SAXS datasets (Mass of full-length gelsolin is 83 kDa). Calculated Mass at temperature X = 83* [I_0_ value at X]/[I_0_ value at 10 °C].

The radius of gyration (R_G_) values calculated from the slope of the linear region of the Guinier approximation for the samples at different temperatures have been plotted as a function of the temperature (Fig. 2E). The analysis revealed that gelsolin molecules in solution at a concentration of 8 mg/ml had an R_G_ value of ~3 nm in temperature range of 10–25 **°**C, which increased marginally to 4 nm by 40 **°**C, and then R_G_ values increased to higher numbers rapidly. In the samples, above 60 °C, whitish flocculants could be seen suggesting initiation of protein aggregation. Thus, the R_G_ values estimated for gelsolin molecules at 60–80 °C had a higher degree of uncertainty (*indicated as red circles* in Fig. 2E). Any case, presuming a two-state transition profile from globular to aggregated state, the mid-point of transition of R_G_ values was about 60 **°**C. Knowing that protein association in most cases is concentration dependent, variable temperature SAXS experiments were repeated with samples having gelsolin concentration from 2–10 mg/ml. In all concentrations, R_G_ values were close to 3 nm in the temperature range of 10–25 **°**C before increasing modestly by 35–40 **°**C followed by a rapid increase. Variation in the R_G_ values indicated that mid-point of transition was directly proportional to the concentration of gelsolin in samples (inset Fig. 2E; please see complete image of native PAGE in Supplementary Fig. 1). This observation supported that more energy is required to shift the population of gelsolin molecules from lower dimensions of closed conformation to increased dimensions of open conformation.

Additionally, using the SAXS datasets, ensemble optimization method (EOM) calculations were done to assess the conformations accessible to proteins in solution (Fig. 2F). Against a random pool of structures, the program computed R_G_ values of conformations which can represent acquired SAXS profile from the ensemble in solution. The distribution of R_G_ values indicated that full-length gelsolin adopted closely related conformations at temperatures 10, 20, 25, and 30 °C. For datasets acquired at 35 °C, a small “tail” of population was seen with higher R_G_ values (please see the inset in the Fig. 2F). Interestingly, employing the same protocol, the SAXS data set acquired for gelsolin at 40 °C suggested two populations. One population was seen close to 3.7 nm and another around 4.2 nm. Average R_G_ values calculated from EOM calculations are compared with the R_G_ values computed from Guinier approximations in Table 2. It is important to mention here that the differences are within the range of error from both modes of calculations. Any case, EOM calculations indicated that gelsolin molecules adopt mainly one set of closely related conformations in the datasets, and there is a gradual increase their average size with increase in temperature.

**Table 2:** A comparison of the radius of gyration (R_G_) values computed for the predominant scattering species in the samples of gelsolin protein at different temperatures as determined from Guinier approximation and ensemble optimization method (EOM) are tabulated below.

**Temperature** (**°C**)	**Radius of Gyration** (**R**_**G**_)		**Difference**
	**Guinier Analysis** (**nm**)	**EOM Analysis** (**nm**)*	
10	3.19 ± 0.23	3.56 ± 0.37	0.38
20	3.24 ± 0.20	3.59 ± 0.30	0.35
25	3.36 ± 0.27	3.62 ± 0.35	0.26
30	3.68 ± 0.23	3.65 ± 0.36	0.02
35	3.71 ± 0.29	3.69 ± 0.29	0.02
40	3.99 ± 0.23	3.90 ± 0.26	0.09

*EOM computes values in Å, and they are converted into nm for comparison. The average and standard deviation values were computed from the output for individual set.

**Figure 3:**
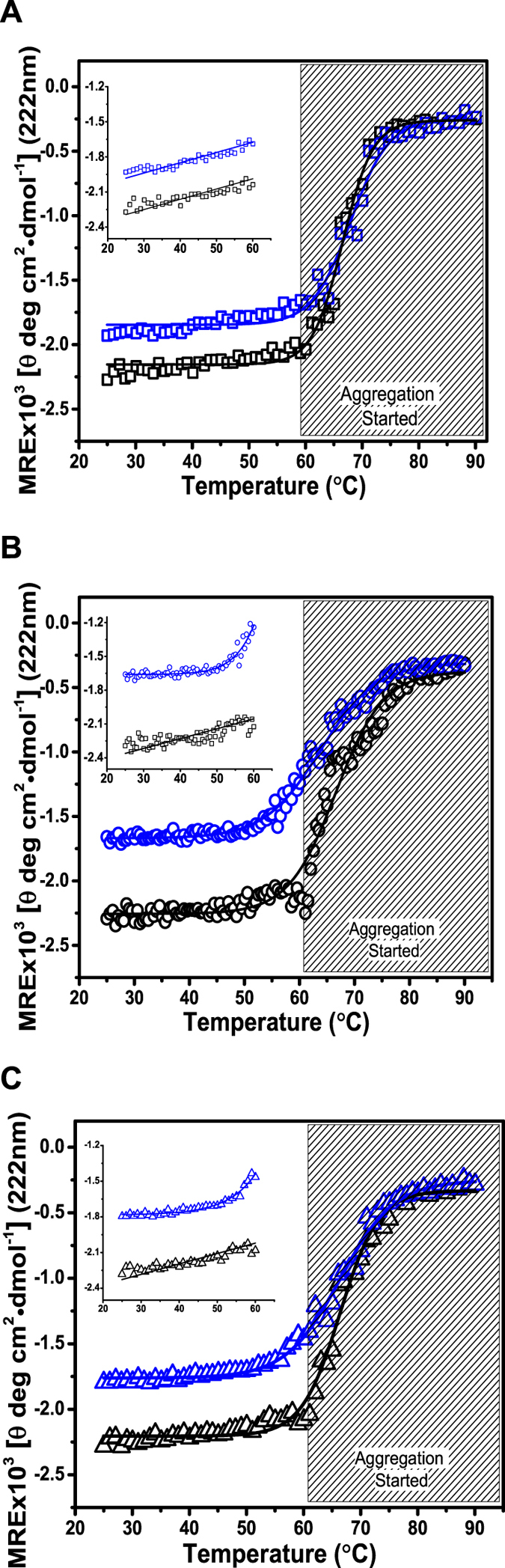
The variations in the mean residue ellipticity (MRE) values at 222 nm in variable temperature CD experiments are plotted here (blue and black squares represent data from gelsolin samples, 0.5 and 1 mg/ml, respectively). The gray shaded zone indicates samples at which whitish aggregation was visible. (**A**) Buffer having 1 mM free Ca^2+^ ions and pH of 8, (**B**) buffer lacking any free Ca^2+^ ions and pH 5, and (**C**) buffer lacking any free Ca^2+^ ions, and pH 8.

To answer whether the heat-induced increase in dimensions of gelsolin molecules were accompanied with loss in secondary structural content, variable temperature CD experiments were done (Fig. 3). Experiments were carried out with gelsolin samples with concentration 0.5 and 1 mg/ml in buffers containing 1 mM free Ca^2+^ ions, no free Ca^2+^ ions but pH of 5 and no free Ca^2+^ ions and pH of 8 to see change in ellipticity values as a function of temperature. In all three conditions, as in SAXS experiments, protein aggregation was visible from 60 °C onwards, and it correlated with earlier reports^9, 10^. Considering the data prior to initiation of aggregation *i*.*e*. below 60 °C, the slow decrement in secondary structural content had a mid-point close to 45 °C for all three conditions (insets in Fig. 3). Also, in correlation with SAXS data based experiments, the sample with lesser protein concentration showed onset of loss in secondary structural content earlier. The relevant conclusion from the CD experiments was that the increased dimension of gelsolin seen from SAXS data analysis capable of depolymerizing F-actin does not accompany a significant change in the secondary structural content.

### SAXS data Guided Shape Reconstruction

**Figure 4:**
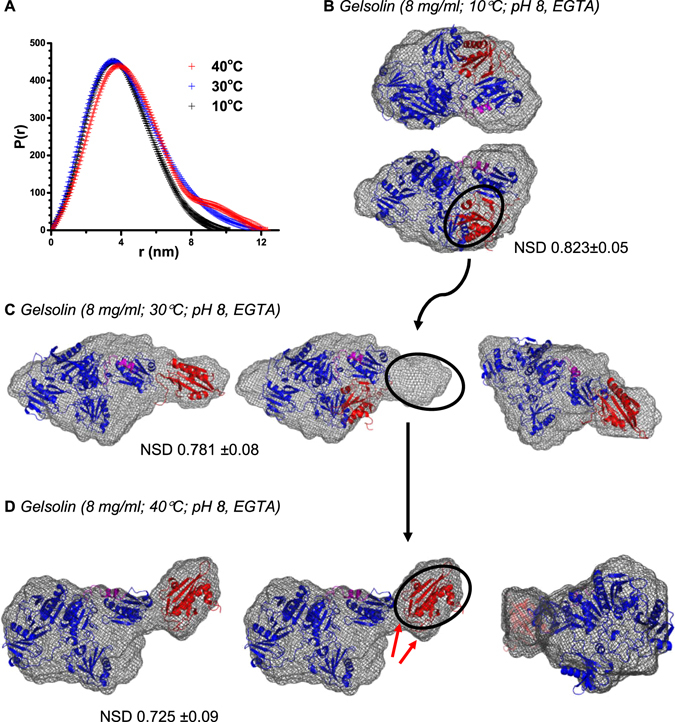
(**A**) The P(r) profiles computed using SAXS data profiles collected for 8 mg/ml gelsolin shown in Fig. 2A are plotted here for temperatures 10, 30 and 40 °C. (**B**–**D**) The SAXS data based dummy residue model solved for gelsolin protein at temperatures 10, 30 and 40 °C are presented. The dummy residues are shown as grey mesh. As discussed in main text, for each SAXS data based model, either crystal structure of Ca^2+^-free gelsolin or its simplistic reoriented model was used to assess the shape rearrangements seen in the SAXS data based dummy atom models. In all structural models used with SAXS data based models, the G1 domain and g1–g2 linker, and the C-tail latch are depicted in red and magenta coloured ribbons, respectively. Additionally, the black circle drawn highlights the location of G1 domain and the arrows between different panels track its repositioning as a function of temperature.

Pairwise distance distribution function (PDDF), P(r) is the frequency distribution of interatomic vectors which best-represents the scattering profile emerging from the molecules in solution. In this work, all the PDDF calculations were done in automated manner considering monodisperse globular nature of the scattering species. Probability of finding vectors corresponding to 0 nm and a dimension equal to the longest vector in the scattering shape were considered to be zero. As described above, I_0_ values from Guinier analysis supported that gelsolin molecules remained primarily monomeric till 40 °C, so we computed P(r) curves from samples (8 mg/ml) at 10, 30 and 40 **°**C are shown in Fig. 4A. Moreover, the temperature induced F-actin depolymerizing activity of gelsolin were maximum in the range of 35–40 °C, thus modeling the scattering shapes of gelsolin in solution at 30 and 40 °C became more relevant. Using the P(r) profiles and I(Q) profiles, scattering shape of the gelsolin was modelled at 10, 30 and 40 **°**C in buffer having pH 8 and 1 mM EGTA or under Ca^2+^-free conditions. The dummy atom model computed for 10 and 20 **°**C compared well with the crystal structure of Ca^2+^-free gelsolin (PDB ID: 3FFN one chain) (*model of 20* °C *is not shown*) (Fig. 4B). *The average normalized spatial disposition* (*NSD*) *value for the ten models are mentioned next to each SAXS data based model*. NSD values close to 1 support that all individual models resemble each other and can be reliably averaged.

The model solved for gelsolin at 30 **°**C showed that the gelsolin molecule appears to open from one side (Fig. 4C) which opened further at 40 **°**C (Fig. 4D). Being aware that opening of the G1 domain of gelsolin makes it competent to bind actin and interfere in polymerization pathway, the G1 domain was highlighted in the model placed inside SAXS based envelope model of gelsolin at all temperatures (red lines). The C-terminal helical latch which holds the G2 and G6 domains have been highlighted as magenta lines in the figures. When crystal structure of Ca^2+^-free gelsolin was positioned in one side of the SAXS based model of gelsolin at 30 °C, the G1 domain of crystal structure remained outside the SAXS based model. At the same time, there was unoccupied volume in the same side of SAXS based model (shown with a circle in middle panel of Fig. 4C). The right hand side panels of the same figure shows that G1 domain abstracted from crystal structure and repositioned into the excess volume of the SAXS data based model leads to better agreement. The model solved for 40 **°**C clearly showed that one domain opened up away from the other five domains. The G1 domain and g1–g2 linker (first 161 residues) clearly fitted in the extended out volume of the SAXS data based model (Fig. 4D). Different views of the models indicated that with heating: 1) the G1 domain opens away from the other five domains which are held together by the Ca^2+^-sensitive C-tail latch, and 2) the extended away G1 domain and g1–g2 linker exposes the actin binding epitopes. These results further concluded that C-tail latch of gelsolin is specifically sensitive to Ca^2+^ ions, and explained the surprising F-actin depolymerizing activity of gelsolin in absence of Ca^2+^ ions upon heating.

### Heat induced shape changes in ΔCT gelsolin and gelsolin lacking G1 domain

**Figure 5:**
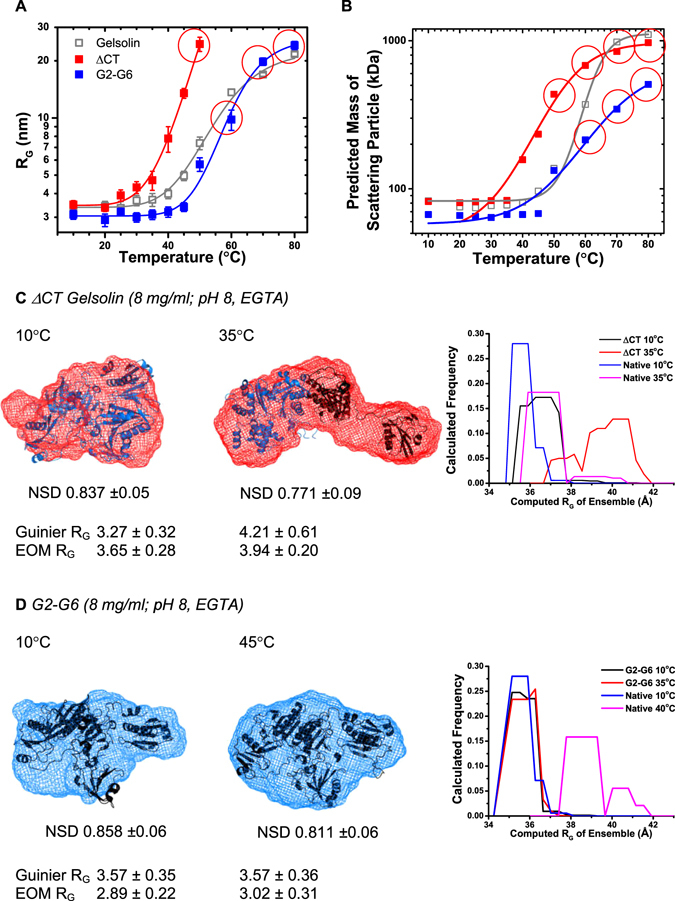
(**A**) The variation in the SAXS data based estimated R_G_ values of ΔCT and G2–G6 gelsolin are shown as a function of time relative to the values computed for full-length gelsolin. (**B**) Variation in the molecular masses of the ΔCT and G2–G6 gelsolin estimated using I_0_ values from Guinier analysis are compared with those estimated for full-length gelsolin. (**C**) SAXS data based dummy residue models of ΔCT gelsolin at 10 and 35 °C (red mesh). Crystal structures are placed inside as described in main text. (**D**) SAXS data based dummy residue models of predominant scattering shape of G2–G6 gelsolin in Ca^2+^ free buffer having pH 8 at 10 and 45 °C (blue mesh). The right panels in both (**C**,**D**) segments show the variation in the R_G_ values of the conformations assessed by EOM calculations.

To further confirm the above conclusions that upon heating only the G1 domain of gelsolin opens up away from the other five domains as the latter are held together by the Ca^2+^ ions sensitive C-tail latch, we studied temperature dependent SAXS profiles of two additional constructs of gelsolin: (1) ΔCT gelsolin – gelsolin lacking the C-terminal latch (sequence terminated at S728 followed by six Histidine tag), and (2) G2–G6 gelsolin – gelsolin lacking G1 domain (the construct started from L162 preceded by a methionine). Variable temperature SAXS data was acquired on both the proteins at concentrations close to 8 mg/ml, and variations in the R_G_ values were plotted as a function of temperature (Fig. 5A). Analysis showed that dimensions of ΔCT gelsolin increased rapidly with increase in temperature as compared to native gelsolin. On the other hand, dimensions of G2–G6 gelsolin remained unchanged up to slightly higher temperatures than for native gelsolin. As done for the full-length gelsolin, molecular masses of the truncated gelsolins were calculated using the I_0_ values estimated from Guinier approximations of the SAXS data at different temperatures (Fig. 5B). R_G_ and I_0_ value based molecular masses estimated from samples indicating temperature induced aggregation or whitishness have been indicated with red circles in Fig. 5A,B. All analyses of the SAXS data supported that while G2–G6 version of protein was resistant to temperature induced changes including association than native protein, the tail-less entity, ΔCT opens up and associates at relatively lower temperature.

Taking cue from the calculated mass of the predominant scattering entity in solution, SAXS data based models were generated for ΔCT and G2–G6 at temperatures 10 and 35 **°**C, and 10 and 45 **°**C, respectively (Fig. 5C,D). NSD values of the ten models solved and averaged for the scattering based shapes are reported in the figures. Solution shape of ΔCT at 10 °C indicated a compact shape with slight opening from one side. Placement of single chain of crystal structure of Ca^2+^-free gelsolin showed that all the six domains of gelsolin can fit inside the overall shape of the SAXS based envelope model (Fig. 5C left panel). Interestingly, the SAXS data based model of ΔCT gelsolin at 35 °C showed an open shape where crystal structures N- and C-terminal halves of gelsolin could be fitted inside the volume of SAXS data based model (Fig. 5C centre panel, black ribbon; G1–G3 from PDB ID: 1RGI, and blue ribbon; G4–G6 from PDB ID: 1H1V). This overlay supported that in absence of C-tail latch, all six domains of gelsolin open-up merely by heating. The role of C-tail latch could also be seen in the compact shape of SAXS data based model of G2–G6 gelsolin at temperatures 10 and 45 **°**C, where a simple model of G2–G6 could be created by merely deleting G1 domain and g1–g2 linker from the crystal structure of Ca^2+^-free gelsolin fitted very well inside the SAXS data based model’s shape profile (Fig. 5D, black ribbon PDB ID: 3FFN one chain).

EOM calculations were performed for ΔCT and G2–G6 gelsolin using their SAXS profiles obtained at 10, and 35 and 45 °C, respectively (Fig. 5C,D right panels). In comparison with full-length gelsolin, ΔCT protein appeared to adopt a wider range of conformations at 10 °C. At 35 °C, ΔCT adopted multiple conformations with major population close to 4 nm. At same time, EOM calculations indicated that G2–G6 adopted closely related conformations about 3 nm at 10 and 45 °C. For comparison, we plotted the calculations for full-length gelsolin done using SAXS profile acquired at 40 °C which highlights the conformational stability of G2–G6 at increased temperatures. Additionally, the R_G_ values calculated from EOM calculations for both proteins are mentioned below the figures and compared with values observed from Guinier approximations. Together, these set of results confirmed that G1 domain opens up away from other five domains since they are “lock-up” by the C-tail latch which cannot be loosened by temperature alone.

### Reversibility of heat induced Opening of Gelsolin

**Figure 6:**
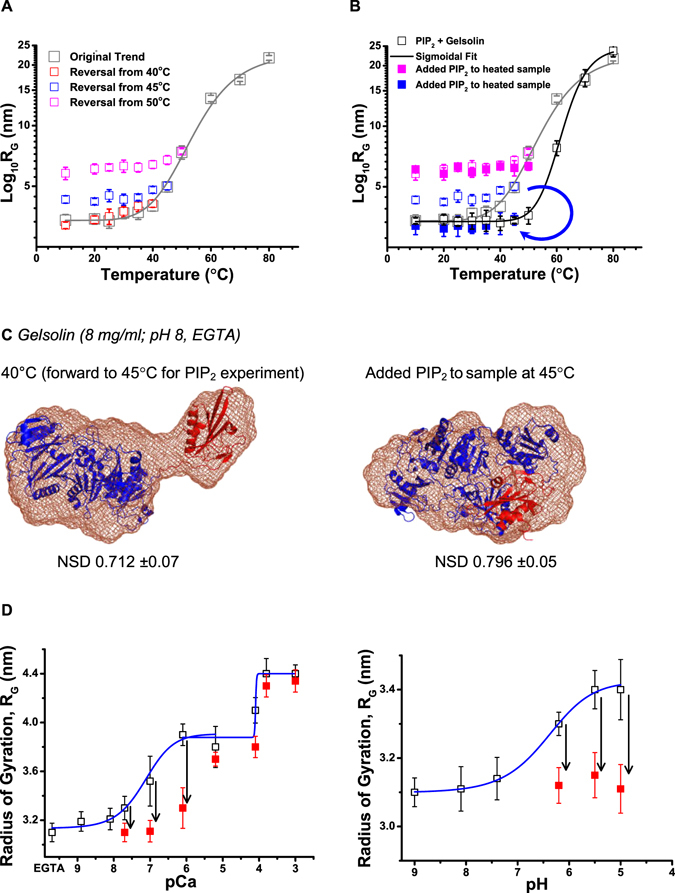
(**A**) Variation in R_G_ values of gelsolin molecules in absence of PIP_2_ as tracked by Guinier analyses of the SAXS datasets obtained at different temperatures are shown. The data here shows whether the R_G_ values decrease upon lowering the sample temperature or not. (**B**) Computed R_G_ values from SAXS datasets obtained from samples of gelsolin mixed with PIP_2_ are presented here as function of temperature. The blue arrow highlights the PIP_2_ addition induced decrement in R_G_ values in gelsolin samples pre-heated to 45 °C. (**C**) SAXS data based scattering shape solved for gelsolin molecules at 40 and 45 °C but without and with PIP_2_ in solution, respectively are shown here (dummy residues shown as brown mesh). As detailed in discussion section, models were placed inside SAXS data based shape profiles for comparison. (**D**) The panels show the variation in R_G_ values of gelsolin value as a function of free Ca^2+^ ions (left) or low pH in buffer (black open squares). The filled red squares show the R_G_ values of the samples having same amount of Ca^2+^ ion or pH in buffer, plus PIP_2_. The black arrows highlight decrement in the R_G_ values upon addition of PIP_2_.

Quick query was raised if the temperature induced opening of gelsolin is a reversible phenomenon since previous characterizations of gelsolin under Ca^2+^-free conditions and at pH 8 showed a compactly packed shape^2, 3^? To explore this, sample of gelsolin (8 mg/ml) in buffer lacking free Ca^2+^ ions and having pH 8 was heated to different temperatures and then the temperature was brought back to 10 **°**C, while acquiring SAXS data at different temperatures. Guinier analysis provided the R_G_ values of scattering particles which indicated that gelsolin molecules can reversibly close if they are heated up to 40 **°**C (Fig. 6A). Samples heated to slightly higher temperatures (45 and 50 **°**C) did not show decrement in shape parameters upon cooling indicating irreparable or irreversible changes are initiated in the gelsolin proteins at these temperatures. Earlier, we had seen an increase in apparent molecular masses of the molecules at these temperatures which indicated some extent of association. In summary, our data indicated that gelsolin molecules open-up at temperatures close to physiological ones which somewhat correlates with enhanced activity reported earlier^6^.

*If the above observations are true in biology then gelsolin may never be inactive as even in absence of Ca* ^2+^ *ions or low pH*, *gelsolin can achieve an actin binding shape*, *and thus interfere in actin assembly process*. Situation can get further complicated since there would be a very likely interplay of temperature and available Ca^2+^ ions or low pH in surroundings of gelsolin molecules. As published before by us, in low pH buffer significantly less amount of Ca^2+^ ions were required to open up the full gelsolin molecule as the C-tail latch is sensitive to Ca^2+^ ions only^3^. In this work, we showed that C-tail latch remains unaffected by temperature, and cutting off C-tail latch allows temperature alone to open-up all domains of gelsolin. We propose that physiological temperature in conjunction with the low levels of Ca^2+^ ions (below 0.1 μM^2^) would be able to completely open gelsolin molecules for their actin assembly regulation function. Outside cells, in plasma, large concentration of free Ca^2+^ ions (~1 mM) would invariably activate gelsolin into a completely open shape^11^. In other words, considering the fact that gelsolin can be activated by Ca^2+^ ions, and/or low pH, and/or (now) physiological temperature, it implies that gelsolin would always be constitutively active both inside cells and outside, unless there is a molecule which deactivates it, fully or partially.

### PIP_2_ deactivates or closes partially open gelsolin

In literature, phosphatidylinositol 4, 5-bisphosphate (PIP_2_) was shown to deactivate gelsolin molecules though no direct structural evidence is available to date^12, 13^. Gel filtration and intrinsic tryptophan fluorescence experiments provided evidence that gelsolin binds PIP_2_ and their affinity is increased by the presence of µM levels of free Ca^2+^ ions and the need for Ca^2+^ ions is reduced upon lowering of buffer pH^13, 14^. Biochemical data have shown that PIP_2_ predominantly binds to the N-terminal half of gelsolin, a portion which is highly conserved amongst the gelsolin superfamily, and to date only one structure (NMR based) is available which shows how the peptide corresponding to residues 150–169 of gelsolin binds to PIP_2_ (PDB ID: 1SOL)^15–18^. The mechanism of action of PIP_2_ and gelsolin inactivation remains debatable, as one notion is that the PIP_2_ competes with gelsolin bound actin for binding to g1–g2 linker of gelsolin, which gets challenged since PIP_2_ binding epitopes are somewhat occluded in bound actin state^11, 19^. Based on biochemical studies, another mechanism has been composed which suggests that PIP_2_ binding dislodges Ca^2+^, leading to release of bound actin to cytoplasm followed by closure of the g1–g2 linker which brings G1 and G2 in proximity of each other^18–20^. Furthermore, the cells with increased PIP_2_ have an altered non-native phenotype similar to that of gelsolin null phenotype which reflects uncontrolled association state of actin inside cells^21^. In summary, PIP_2_ is known to bind the residues in the end of the G1 domain and the g1–g2 linker which become available only when the g1–g2 linker opens up from the compact shape of the inactive gelsolin. Since temperature induced activation of gelsolin also involves extension of g1–g2 linker, the known site for PIP_2_ binding, we decided to explore: (1) if PIP_2_ can deactivate temperature-activated gelsolin and (2) whether these events can be tracked by SAXS data analysis which may explain vital role of PIP_2_ in regulating “apparently always” active gelsolin at least inside cells.

As mentioned in methods, PIP_2_ was added to gelsolin sample and change in the R_G_ values of the protein molecules was tracked using Guinier analysis of the acquired SAXS datasets (Fig. 6B). The R_G_ values of gelsolin+PIP_2_ sample showed a delayed increment compared to gelsolin alone indicating that PIP_2_ somewhat quenched the heat induced opening of the gelsolin molecules. Additionally, when PIP_2_ was added to gelsolin sample pre-heated to 45 **°**C, the R_G_ values decreased from 5 to 3.4 nm supporting that addition of PIP_2_ can close the semi-open shape of temperature-activated gelsolin (please see the open and filled blue squares in Fig. 6B). *Yet the same could not be seen for sample heated to 50 °C* *indicating that the reversal is possible but only close to physiological temperatures* (please see the open and filled magenta squares in Fig. 6B). Shape restoration using SAXS data acquired for sample at 40 **°**C and on way to 45 **°**C before adding PIP_2_ clearly showed semi-open with G1 domain extending away from other five domains. Within this SAXS data based envelope model, G1 domain and g1–g2 linker from crystal structure of N-terminal half bound to actin (PDB ID: 1RGI; red ribbon) and G2–G6 domains from crystal structure of Ca^2+^-free gelsolin (PDB ID: 3FFN chain A; blue ribbon), could be fitted reasonably well (Fig. 6C left). As somewhat expected, the SAXS dataset of PIP_2_+gelsolin at 45 **°**C resulted in a model with stark resemblance to compact inactive Ca^2+^-free gelsolin as seen in the overlay shown in Fig. 6C (right). Overall, we showed that addition of PIP_2_ can induce reversal of semi-open shape of gelsolin to its closed inactive form.

Earlier, by monitoring decrement in F-actin depolymerizing activity PIP_2_ has been shown to deactivate Ca^2+^- or low pH activated gelsolin^12, 22, 23^, but no structural insight is available to date. Taking cue from above experiments, we repeated SAXS based tracking of shape changes in gelsolin molecules as a function of Ca^2+^ ions or low pH (Fig. 6D)^2, 3^. The observed R_G_ values were in good agreement with previous reports and the Ca^2+^ or low pH induced variation followed a three state or two state trends, respectively. In parallel set of samples, PIP_2_ was added, and SAXS experiments were done. Results showed a drop in the R_G_ values of gelsolin molecules upon addition of PIP_2_ (Fig. 6D). In case of Ca^2+^-activated gelsolin, R_G_ values decreased to 3.1 nm (comparable to inactive or compact gelsolin shape) up to a pCa value of 7.1 or 0.1 µM. For higher concentrations, a decrease for visible but it was insignificant after free Ca^2+^ concentrations surpassed 10 µM (Fig. 6D, left panel). Similarly, addition of PIP_2_ to gelsolin samples at pH 6.1, 5.5 and 5 showed decrease in R_G_ values close to those molecules at pH 8 (Fig. 6D, right panel). Modeling of the datasets with PIP_2_+gelsolin showed an envelope shape which resembled structure/shape of inactive compact gelsolin, as seen in Fig. 6C right. These results support that PIP_2_ can deactivate gelsolin molecules under sub-micromolar Ca^2+^ ion concentration or under low pH conditions.

## Discussion

**Figure 7:**
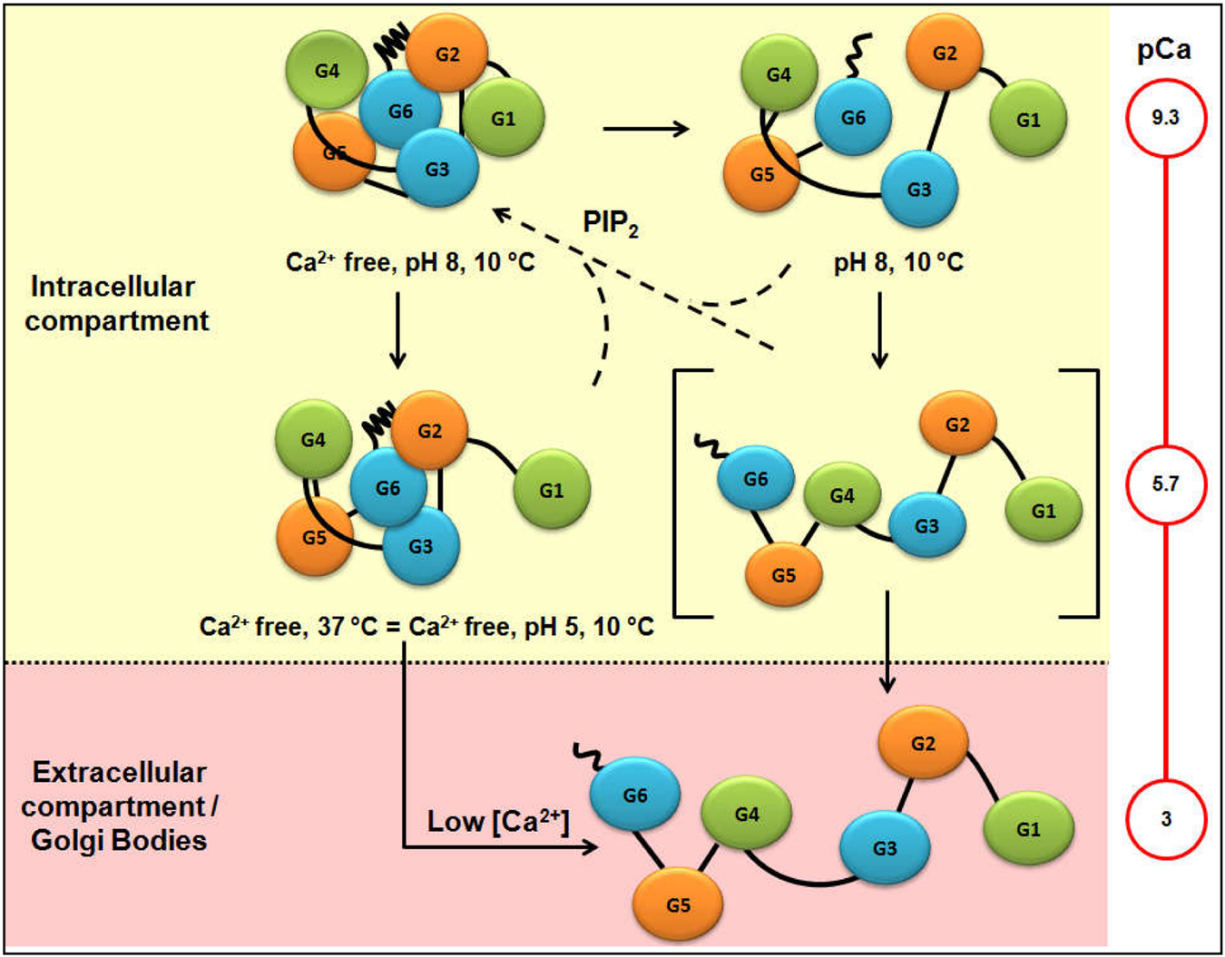
Schematic summary of partially and fully open shapes of gelsolin induced by different physiological regulators of its activity *i*.*e*. Ca^2+^ levels, pH and temperature inside and outside cellular environment. Compilation also sums up how PIP_2_ can revert activation, but only from partially open or activated state.

Shape-function studies in this work brought forth a new physiological activator of gelsolin *i*.*e*. temperature. This is a very basic factor which plays along, if not override other biochemical activators of gelsolin known to date *i*.*e*. Ca^2+^ ions and low pH. Combining with previously published results from our and other groups, it can be summarized that at low temperature all six domains of gelsolin pack tightly under Ca^2+^ free conditions at pH close to physiological value (Fig. 7). Trace amounts of Ca^2+^ ions can open the protein under same conditions by opening the C-tail latch which is only sensitive to Ca^2+^ ions. In absence of Ca^2+^ ions, pH close to 5 (pI of gelsolin is 5.7) can induce partial opening of gelsolin where G1 domain moves away from C-tail locked G2–G6 domains. We showed in this work that in absence of these two factors, increase in temperature to 35–40 °C can also open and stabilize G1 domain away from the other five locked domains analogous to that induced by low pH. Based on our previous work and complementary work from other groups, there was a query that in physiology, in absence of Ca^2+^ ions or low pH, gelsolin adopts an inactive resting shape? Our present shape information in conjunction with results reported earlier indicate that very likely, at physiological temperature, intracellular gelsolin exists in this partially open form and the inactive compact shape may never exist except in availability of PIP_2_ which binds to g1–g2 linker and induces shape changes which bring G1 domain close to G2 domain. In contrast, the high concentrations of free Ca^2+^ ions dictate the predominant shape of the gelsolin molecule. These scenarios are possible in plasma where free Ca^2+^ concentration is close to 1 mM and under injury or stress conditions leading to rise in intracellular Ca^2+^ concentration. Essentially, the high Ca^2+^ ion concentration (much higher than local PIP_2_) will lead to the fully active state of gelsolin due to additional factors of low pH or temperature which will enable rapid remodelling of F-actin assembly where the latter is essential for many cellular functions. Another scenario could be the release of Ca^2+^ ions from the endoplasmic reticulum (ER) membranes, which is released only when a signal is received in the form of inositol triphosphate (IP_3_) molecule. Interestingly, IP_3_ molecule is the product of PIP_2_ hydrolysis by Phospholipase Cγ1 (PLCγ1)^24^ which suggests a correlation between local PIP_2_ and Ca^2+^ levels. Gelsolin and its activation by low pH was discussed by us in view of lowering of pH during apoptosis^3^. We found one publication which linked apoptosis and IP_3_-linked mitrochondrial Ca^2+^ signals^25^ which indicates that PIP_2_ and Ca^2+^ and pH could be interconnected in regulating gelsolin’s function.

Interestingly, PIP_2_ is primarily located at the cell membrane as well as perinuclear spaces, however the PIP kinase mediated synthesis of PIP_2_ in plasma membrane is also reported^26, 27^. It is also proposed that certain proteins might serve as “buffers” which bind and sequester PIP_2_ in the plasma, and subsequently concentrate PIP_2_ in lateral membrane domains^26^. Gelsolin has been suggested to weakly bind PIP_2_ which suggests that PIP_2_ may get released at some point. As of today, it is clear that inactive gelsolin cannot bind PIP_2_, but there is no data which supports a differential affinity of PIP_2_ with open *vs*. closed shape of gelsolin. Thus, the precise mechanism of release of PIP_2_ remains unclear. For now, we opted to use SAXS data analysis and modeling to interpret the unexpected F-actin depolymerization results as a function of temperature as this technique is not limited by the need for a diffraction quality crystal or extensive timelines for labelling and/or imaging based methods. Moreover, SAXS experiments can aid in tracking shape changes in protein molecules albeit experiments are planned properly and results are supported by orthogonal approaches as we did by developing and studying mutant proteins. Considering the new queries raised and their implications in biology, we intend (and invite other researchers) to extend these findings in cell line based experiments and understand how the shape changes affect the signalling pathways associated/dependent on gelsolin. Thus, temperature plays a key player inside cells in activating shape of gelsolin which in turn regulates multiple functions related to assembly state of actin, and flux of available PIP_2_ (which in turn is regulated by Phospholipase C) controls active state of gelsolin by specifically closing the g1–g2 linker.

## Methods

### Gelsolin(s) for experiments

Different gelsolins used in this work: full-length gelsolin, gelsolin lacking C-tail latch (ΔCT), and gelsolin lacking G1 domain (G2–G6) were expressed in vectors and cells as published before refs 3 and 5. Briefly, all proteins had His-tag at their C-terminals (except for full length gelsolin where His-tag is present at the N-terminal) and first round of purification from cell lysates involved Ni-NTA based affinity protocols. Subsequently, the imidazole in the eluted proteins were removed by dialysis, followed by concentration of the proteins and further purification to homogeneity by FPLC as reported before. Purified proteins were concentrated and stored in Tris-Cl buffer, pH 8 containing 2 mM EGTA at concentration close to 8 mg/ml in −20 °C. Prior to all experiments, purity (and stability) of the gelsolins were confirmed by the expected migration pattern of the single band in 10% SDS-PAGE. Along with, mass of the proteins were confirmed by MALDI-TOF. Concentrations of proteins for SAXS and functional assays were estimated by using their calculated absorbance value at 280 nm. Calculated molar extinction coefficients of gelsolins using their primary structures including tag were used for estimation of protein concentrations (http://web.expasy.org/protparam/).

### Pyrene labelled F–actin depolymerizing assay

The rate of decrement in fluorescence of pyrene–labelled F–actin in presence of gelsolin was used to determine the ability of gelsolin to depolymerize actin filaments as done before^8^. Briefly, pyrene labelled G–actin was converted to F–actin by adding 3 mM MgCl_2_ and 100 mM KCl in presence of gelsolin (in Actin:Gelsolin molar ratio of 500:1) followed by overnight incubation at 20 °C. To measure depolymerization capability, stock of pyrene-F–actin (9.88 µM) was diluted to 100 nM in F–buffer (0.2 mM Tris HCl, 0.2 mM CaCl_2_, 0.2 mM ATP, 0.5 mM 2–mercaptoethanol at pH 8). For activity assays under Ca^2+^-free conditions, 0.2 mM CaCl_2_ in F–buffer was replaced with 0.2 mM EGTA. To understand the effect of temperature on the activity of gelsolin, vials containing 50 μl of solution of gelsolin (1.4 mg/ml or 17 μM) was heated at the desired temperature (in the range of 10 to 80 **°**C) for 30 minutes MyGene^TM^ Series Peltier Thermal cycler (LongGene Scientific Instruments Shanghai, China). Subsequently, the protein solution was diluted to 1.8 μg/ml (20 nM) in buffers of respective pH, kept at 25 °C and used for depolymerization assays which were done at 25 °C. It is worth noting here that same gelsolin concentration was used for observing/comparing depolymerization ability of gelsolin as a function of Ca^2+^ ions or pH in buffer. The fluorescence assays were performed in a 96 well flat bottom FlouroNunc plates. In each well, total reaction volume was close to 200 µL which included gelsolin and F–actin in a molar ratio of 1:10 (Gelsolin: F–actin). Importantly, F–actin was added last in the wells and readings were taken within 15–20 seconds of final addition. For measurements, excitation wavelength was kept at 365 nm and emission was recorded at 407 nm using Tecan plate reader with i-control software (Männedorf, Switzerland). The numbers of flashes were kept at 25 with an integration time of 20 µs. Each sample was assayed in duplicates, and the average of reading of both the samples was taken as final readout.

### SAXS data acquisition, processing and analysis

To study the effect of varying temperature on the global shape of gelsolin, SAXS datasets were collected using SAXSpace instrument (Anton Paar GmbH, Graz Austria). At temperature intervals of 5 or 10 **°**C, SAXS data was collected on gelsolin solution (about 30 μl) and matched buffer from 10 to 80 **°**C. Line collimation was used for incident X-rays on sample filled in a quartz capillary of 1 mm diameter for 30 minutes. Scattered X-rays were recorded on a 1D CMOS Mythen detector (Dectris Switzerland). SAXS datasets for sample and buffer were processed using SAXStreat and SAXSquant softwares. Using the beam profile, the data collected on line collimation was desmeared to represent scattering arising from point collimation. Intensity of scattering, I(Q) was obtained as a function of Q, where Q was considered as 4 π sin θ/λ. All processing conditions were identical for all datasets, and I(Q) profile of the respective buffer was subtracted from the I(Q) profile of the solution to obtain SAXS profile arising from the protein molecules in solution. SAXS data profile(s) of gelsolin at different temperatures were analyzed using ATSAS 2.7.1 pipeline of programs to obtain information about scattering size and shape of the protein molecules at that temperature. Briefly, Kratky analysis, Guinier approximations and automated estimations from probability distribution of interatomic vectors were done using the suite of programs.

### Ensemble Optimization Method (EOM) Calculations

Using the experimental SAXS data as reference and the EOM program in the ATSAS 2.7.1 suite, calculations were done to estimate the R_G_ values of the various conformations accessible to the gelsolin molecules at different temperature. This genetic algorithm based program presumes the observed shape profile from the scattering species in solution to be a weighted average of ensemble of conformations accessible to the molecule in solution, and attempts to describe the experimentally acquired SAXS profile as an ensemble of models with varying R_G_ values. Earlier, it has been successfully applied for flexible proteins to map the R_G_ values of the conformations accessible to proteins in solution^28^. We have applied this protocol to address ensemble of conformations accessible to a well-known inherently disordered protein, Calmodulin^29^. For this work, against the random pool of structures, optimized ensemble of 5000 conformers were generated by performing 100 independent runs of 50 conformers each. Using the SAXS profiles of the full-length gelsolin at different temperatures, and SAXS data of ΔCT gelsolin and G2–G6 gelsolin at different temperatures, EOM calculations were done to estimate R_G_ values of the conformations in the ensemble.

### Circular dichroism (CD) measurements

To gain insight into the secondary structural content in gelsolin molecules and their change as a function of temperature, CD experiments were carried out using Jasco spectrophotometer 815 (Tokyo Japan). The temperature for the experiments was controlled with a Peltier type temperature control system, model PTC-424S. A quartz cell with 0.2 cm path length was used and spectra were recorded from 205 to 250 nm at intervals of 1 nm. For each sample and buffer, 5 scans were averaged and final spectral profile of gelsolin was obtained by subtracting profile of the buffer. Temperature scans were done with temperatures starting from 25 to 90 **°**C, with a temperature ramp of 3 °C/min. From the complete scans, ellipticity values recorded at 222 and 218 nm were plotted to compute melting temperature, T_m_ or the temperature at which half of the α-helical or β-sheet content was unfolded during the acquisition time of the data.

### Native PAGE characterization of heated gelsolin samples

The continuous native PAGE was performed as described earlier^30^, albeit with slight modifications. Briefly, a 10% non-denaturing gel was cast (pH 8.8), and this gel was pre-run at 10 V/cm for one hour at 4 °C before loading samples. After pre-run, the electrode buffer was discarded and replaced with fresh buffer. Samples of FPLC purified gelsolin at concentration same as that used for SAXS experiments (8 mg/ml) were individually heated from 10 to 80 °C for 30 minutes and cooled back to 25 °C in a thermal cycler. From these vials 2 μL (16 μg per lane) of protein mixed with 2x non-denaturing gel loading dye (pH 8.8) and loaded in the wells of on the native gel. Post-run, the gel was stained with 0.25% Coomasie Brilliant Blue R – 250, followed by de-staining and image was captured using AlphaImager (ProteinSimple, California, USA).

### SAXS data based shape restoration

Scattering shapes of gelsolin or versions at different temperature point were reconstructed using chain-like ensemble modeling program GASBOR in reciprocal space using SAXS I(Q) profile and results from its pair wise distribution of interatomic vectors^31^. Dummy aspartic amino acid residues equal to mass of the protein molecules were considered to model the shape of gelsolin and its variants. Ten models were generated for each dataset, and they were averaged using DAMAVER suite of programs. Essentially, for each set, the models were superimposed over a reference model by aligning inertial axes using SUPCOMB20 program from ATSAS suite, and their NSD values were calculated. Any models with NSD value more than twice the standard deviation is rejected by the averaging program. In this work, none of the models were rejected being very similar to each other in the sets, except one of the ten for ΔCT gelsolin at 35 °C. The averaged models are presented in the Figures, and were compared with crystal and modelled structures. Open source version of Pymol was used for visual analysis of results and figure generation presented in this publication^32^.

### SAXS experiments of Gelsolin −/+ PIP_2_

For this experiment, 1:1 molar mixture of gelsolin with PIP_2_ was prepared (gelsolin was 2.8 mg/ml; 35 μM). This mixture was used to acquire SAXS data collection in the temperature range of 10–45 **°**C to monitor if any shape changes occur to gelsolin pre-mixed with PIP_2_. Additionally, PIP_2_ was mixed with gelsolin sample (~3 mg/ml) heated to 40 and 50 **°**C, respectively, and the sample was cooled back while measuring SAXS data at different temperatures. The SAXS data from these samples were analyzed as described above.

## Electronic supplementary material

Supplementary Information
