# Supplementary material for: Visualizing Temperature Mediated Activation of Gelsolin and Its Deactivation By Pip2: A Saxs Based Study
Source: Sci Rep. 2017 Jul 5;7:4670. doi: 10.1038/s41598-017-04975-0 (PMC5498565; doi:10.1038/s41598-017-04975-0)
Supplement: Supplementary file 1 — Supplementary Information [file 41598_2017_4975_MOESM1_ESM.pdf]

Supplementary Data

VISUALIZING TEMPERATURE MEDIATED ACTIVATION OF GELSOLIN AND ITS DEACTIVATION BY PIP_2: A SAXS BASED STUDY

MAULIK D. BADMALIA, SHIKHA SINGH, RENU GARG AND ASHISH*
CSIR-INSTITUTE OF MICROBIAL TECHNOLOGY, CHANDIGARH INDIA

**Address correspondence to*: Ashish, CSIR-Institute of Microbial Technology, Sec 39-A, Chandigarh 160036 INDIA Phone: +172-6665472; Fax: 172-2636680; E-mail: ashgang@imtech.res.in

Figure S1

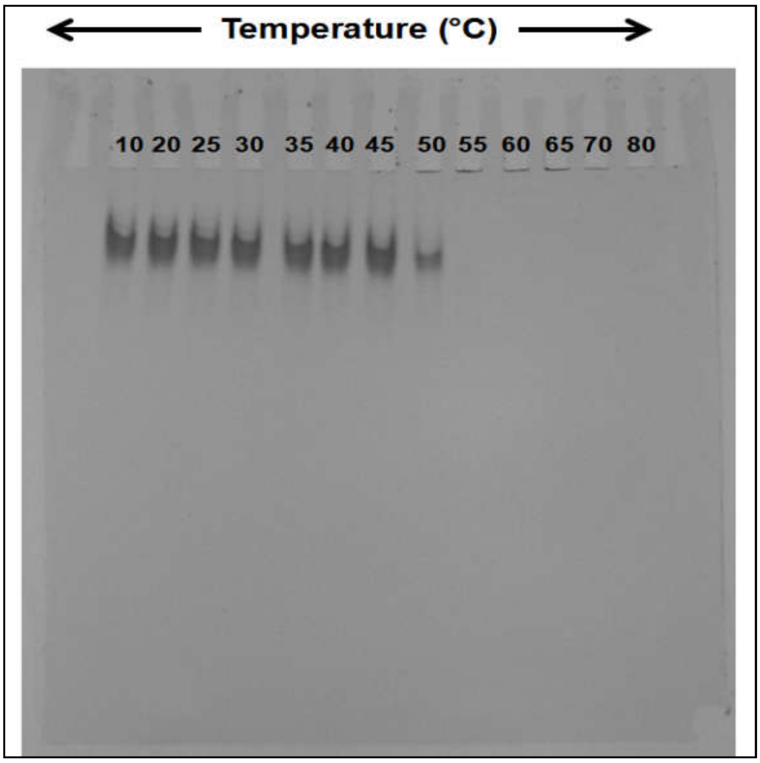

Effect of temperature on gelsolin as characterized on a 10% Native-PAGE.

The samples shown in this gel were prepared and loaded on the Native-PAGE exactly as described in the methods section. This image is the complete image of inset image shown in Figure 2D.
